# High resolution structures of the SARS-CoV-2 N7-methyltransferase inform therapeutic development

**DOI:** 10.21203/rs.3.rs-1370473/v1

**Published:** 2022-03-08

**Authors:** Aneel Aggarwal, Jithesh Kottur

**Affiliations:** Icahn School of Medicine at Mount Sinai; Icahn School of Medicine at Mount Sinai

## Abstract

Emergence of SARS-CoV-2 coronavirus has led to millions of deaths globally. We present three high-resolution crystal structures of the SARS-CoV-2 nsp14 N7-methyltransferase core bound to S-adenosylmethionine (SAM; 1.62Å), S-adenosylhomocysteine (SAH; 1.55Å) and Sinefungin (SFG; 1.41Å). We identify features of the methyltransferase core that are crucial for the development of antivirals and show SAH as the best scaffold for the design of antivirals against SARS-CoV-2 and other pathogenic coronaviruses.

The capping of viral mRNAs is essential for their stability, efficient translation, and evasion of the host immune response and is mediated in SARS-CoV-2 by methyltransferases (MTases) nsp14 and nsp16^1^. Nsp14 methylates the N7 atom of guanosine to generate the ^N7Me^GpppA_2’OH_-RNA or “cap-0” structure, which is then subsequently methylated at the 2’O atom of the initiating nucleotide by nsp16 to make the ^N7Me^GpppN_2’OMe_-RNA or “cap-1” structure^1^. Both nsp14 and nsp16 use SAM as the methyl donor and generate SAH as the reaction byproduct. The methylase activity of nsp14 resides in its C-terminal N7-MTase domain (Fig. 1a)^2,3^. The N-terminal end of nsp14 bears the exoribonuclease domain (ExoN) that is involved in maintaining replication fidelity and requires interactions with the cofactor nsp10^2,4^. The centrality of nsp14 N7-MTase to the SARS-CoV-2 life cycle makes it an attractive target for the development of antivirals, but there is no high-resolution structure of the N7-MTase to guide the development of such inhibitors. Most of the structure-guided efforts have thus depended on crystal structures of nsp14/nsp10 from SARS-CoV^5,6^, solved to low 3.2 to 3.4 Å resolution^2^. Although the SARS-CoV-2 nsp14/nps10 has recently been imaged by cryo-EM (including in complex with other RTC components), the resolution of these cryo-EM structures is limited to 2.5 to 3.9 Å and they do not capture interactions with bound SAM, SAH or SFG (a general MTase inhibitor)^7,8^.

To elucidate the structural determinants of SAM, SAH and SFG binding to the SARS-CoV-2 nsp14/nsp10 complex, we employed fusion protein-assisted crystallization^9,10^ and determined high resolution crystal structures of the nsp14 N7-MTase-TELSAM fusion (Fig. 1b) in complex with SAM, SAH, and SFG (Supplementary Table 1).

The MTase core in the three structures is nearly identical, superimposing with RMSDs between 0.085 to 0.09 Å for 187 Cα atoms, showing it to be essentially invariant when bound to SAM, SAH or SFG (Fig. 1c). The MTase core consists of an atypical Rossmann fold, composed of a central five stranded β-sheet (β1’, β2’, β3’, β4’ and β8’) instead of the seven stranded β-sheet (β1–β7) typically associated with class I MTases^11^, including those from most viruses. Helices α1’, α2’, α3’ and αC, β-strands βA and βB, and a loose Zn^2+^ coordinated substructure are located on one side of the β-sheet, and two short helices αA and αB on the other (Fig. 1b). SAM, SAH and SFG are located at the C-terminal ends of strands β1’, β2’, β3’, and are cradled by loops between β1’ and β2’, β2’ and αA, and β3’ and β4’ (Fig. 2a–c).

In the full length nsp14 structures^2,7,8^, a characteristic of the MTase fold is a “hinge” region composed of a three stranded β-sheet (β5’, β6’and β7’; residues 402–433) and an interdomain loop (residues 288–299) that precedes the MTase core (Supplementary Fig. 1a–c). The β-sheet extends from the MTase core and interacts with the ExoN domain and flexibility of the hinge has been suggested to allow for the movement between the MTase core and the ExoN domain^12^. Intriguingly, this β-sheet is disordered in our three structures, suggesting that its interactions with the ExoN domain are required for its folding and stability (Supplementary Fig. 1a). Excluding the hinge region, the SARS-CoV-2 and SARS-CoV nsp14 N7-MTase cores superimpose with an RMSD of 0.67 Å for 183 Cα atoms. The most significant difference is in residues 467 to 482, which fold into helix αC and β-strand βB in SARS-CoV-2 nsp14 (Supplementary Fig. 1d).

The adenine base of SAM, SAH and SFG is ensconced in a cavity formed by the Ala353, Phe367, Tyr368, Cys387 and Val389 side chains, while the N1 and N6 atoms make hydrogen bonds with the backbone amide and carboxyl groups of Tyr368, respectively (Fig. 2a–c). The ribose sugar makes direct hydrogen bonds with the Asp352 side chain, as well as water mediated interactions with both the Gln354 side chain and main chain. Asp352 is conserved in coronaviruses and its mutation to alanine in SARS-CoV has been shown to abrogate the N7-MTase activity^2,3,13^. The tail portion is fixed by numerous interactions, including direct hydrogen bonds with the Arg310 side chain and the Gly333 and Trp385 main chain atoms, as well as intricate water mediated interactions with the Gln313 and Asp331 side chains and the Ile332 and Trp385 main chains (Fig. 2a–c). In addition, the Pro335 ring is involved in van der Waals contacts with the non-polar portion (atoms Cβ and Cγ) of SAM/SAH/SFG. Arg310 and Asp331 are conserved in coronaviruses and their mutation to alanine in SARS-CoV has been shown to abolish N7-MTase activity^2,3^. Thus, even though Asp331 is not involved in a direct hydrogen bond with the ligand, its interaction via a water molecule makes it crucial for N7-MTase activity^2,3^. Indeed, the entire nsp14 MTase-ligand interface is defined by an unusually large number of well-ordered water molecules that mediate hydrogen bonds between the ligand and the protein (Fig. 2a–c). Many of these are “good waters” in that they bridge the MTase and SAM/SAH/SFG and can be considered as extensions of the MTase amino acids in the SAM/SAH/SFG binding pocket. Their displacement will be energetically unfavorable and will be an important feature to take into account in the design of SAM competitive inhibitors of the SARS-CoV-2 N7-MTase.

All of the amino acids at the interface are conserved in the SARS-CoV nsp14 N7-MTase. The crystal structure of SARS-CoV nsp14/nsp10 with SAM captured a subset of the interactions we observe here (Supplementary Fig. 2a, b), but some key interactions such as between Arg310 and the terminal carboxylate group of SAM were not observed, possibly because of the moderate resolution of the structure (Supplementary Fig. 2a). Also, the configuration of the bound SAM is different, wherein the donor methyl group points in the opposite direction to what we observe here (Supplementary Fig. 2b). Most importantly, the limited resolution of the SARS-CoV structure did not allow for the observation of water molecules, which form a crucial part of the N7-MTase-SAM interface (Supplementary Fig. 2a).

Curiously, from our isothermal titration calorimetry (ITC) analysis, SFG binds the SARS-CoV-2 nsp14/nsp10 complex with a similar affinity as SAM (*K*_D_ of 4.4μM versus 5.7μM), but SAH binds ~ 20-fold better (*K*_D_ of 0.3μM versus 5.7μM) (Supplementary Table 2 and Supplementary Fig. 3). How to explain the higher affinity of SAH compared to SAM or SFG? In the nsp14-MTase_SAM_ structure, the donor methyl group of SAM (attached to its Sδ atom) abuts the Asn386 main chain carbonyl and appears to displace a water molecule that would normally be coordinated to the main chain carbonyl (Fig. 2a). Indeed, in the nsp14-MTase_SAH_ structure, we observe a well-ordered water molecule coordinated to the Asn386 main chain carbonyl at a position that would be incompatible with the methyl group of SAM (Fig. 2b). The entry of this water molecule may provide a partial explanation for the higher affinity of SAH relative to SAM, particularly the more favorable enthalpic contribution to binding (Supplementary Table 2 and Supplementary Fig. 3). It is less clear, however, why SAH would bind better than SFG. The amino group of SFG (attached to its Cδ) makes a direct hydrogen bond with the Asn386 main chain carbonyl and would appear to compensate for the loss of a water molecule (Fig. 2c). Whether this hydrogen bond is less favorable enthalpically than a coordinated water molecule to the Asn386 main chain carbonyl is uncertain at present.

The SARS-CoV-2 N7-MTase is a viable target for the design of antivirals. In particular, the emerging role of histone MTases in cancer has spurred the development of many new SAM competitors^14,15^, some of which have entered clinical trials for various cancer malignancies. One attractive feature of SARS-CoV-2 N7-MTase as a drug target is its high conservation of sequence across other coronaviruses and nearly total conservation of sequence across all the strains of SARS-CoV-2 identified recently, including delta and omicron (Supplementary Fig. 4a, 4b). Thus, the N7-MTase offers the prospect of designing broad spectrum antivirals for both present and future coronavirus outbreaks. Interestingly, we find that the affinity of SAH for nsp14 is substantively (~ 15–20-fold) better than for SAM or SFG; positing SAH as the scaffold of choice for the design of more potent SAM competitors. Notably, the N7-MTase-SAM/SAH/SFG interface also contains a conserved cysteine (Cys387) at 3.9 Å and 4.6 Å from the N7 and N6 atoms of the adenine base, respectively (Fig. 2), allowing for a suitable “warhead” on the adenine base to make a covalent bond with the conserved cysteine. Such covalent inhibitors have previously been designed for other MTases^15^, including one that forms a covalent bond with Cys449 in the active site of protein arginine methyltransferase 5 (PRMT5)^16^.

In conclusion, we present here high resolution structures of SARS-CoV-2 nsp14 N7-MTase bound to ligands SAM, SAH and SFG, as an important step towards the development of antivirals against SARS-CoV-2 and other pathogenic coronaviruses.

## Online Methods

### Full-length nsp14/10 complex purification.

For ITC binding studies, single pRSF-duet-1 plasmid bearing C-terminal 6xHis-tagged full-length nsp14 and nsp10 was transformed into *Escherichia coli* BL21Gold (DE3) cells (Agilent). The cells were grown at 37°C until the culture reached an OD_600_ of ~ 0.5, after which the temperature was reduced to 30°C and ZnCl_2_ added at a final concentration of 20uM. At an OD_600_ ~ 0.8, the temperature was reduced to 15°C and expression of the complex was induced by adding 0.5mM IPTG and incubating for 18 hours. The cells were harvested by centrifugation and resuspended in binding buffer (25mM Tris pH 7.5, 250mM NaCl, 10% glycerol, 0.01% IGEPAL, 25mM imidazole, 10μM ZnCl_2_ and 10mM 2-mercaptoethanol). The cells were lysed by sonication in the presence of EDTA-free Pierce Protease Inhibitor tablets (Thermo Fisher) and 1mM PMSF, and the cell debris clarified by centrifugation. The filtered supernatant was loaded onto a HisTrap HP affinity column (GE Healthcare). The column was washed with binding buffer to remove the non-specific proteins bound to the column and the desired complex was eluted using a binding buffer with 500mM Imidazole. The fractions containing the nsp14/nsp10 complex were concentrated and further purified by size exclusion chromatography using a HiLoad 16/600 Superdex 200 (GE Healthcare) column, pre-equilibrated with 100mM KH_2_PO_4_/K_2_HPO_4_ buffer pH 8.0, 100mM KCl, 0.01% IGEPAL, 5mM 2-mercaptoethanol and 10% Glycerol. The fractions containing pure nsp14/nsp10 complex were concentrated and used for ITC without freezing.

### Protein purification for crystallization.

Our efforts to crystallize various constructs and mutants of the nsp14/nsp10 complex and the N7-MTase domain alone (with and without various expressions tags and protein fusions such as green fluorescent protein (GFP)) were generally unsuccessful. Reports on the fusion of TELSAM with target proteins to improve their crystallization^9,10^motivated us to fuse the nsp14 MTase domain (AA300–527) with TELSAM (AA47–124) with different linkers (A, PA and PAA) and carried out expression and protein purification as follows. The pRSF-Duet-1-smt3 plasmids containing N-terminal 6xHis-SUMO-TELSAM-MTase were transformed into *Escherichia coli* BL21Gold (DE3) cells. The cells were grown at 37°C until OD_600_ reached 0.8, and then the temperature was reduced to 15°C and IPTG and ZnCl_2_ added to final concentrations of 0.5mM and 20uM respectively. The cells were harvested 18 hours post induction and resuspended in binding buffer (25mM Tris pH 7.5, 500mM NaCl, 10% glycerol, 0.05% IGEPAL, 30mM imidazole, 10μM ZnCl_2_ and 10mM 2-mercaptoethanol) in the presence of EDTA-free Pierce Protease Inhibitor tablets (Thermo Fisher) and 1mM PMSF. The cells were lysed by sonication and the filtered supernatant was loaded onto a HisTrap HP affinity column (GE Healthcare). The column was washed with binding buffer containing 1M NaCl to remove non-specific proteins bound to the column. The column was then re-equilibrated with binding buffer and Ulp-Protease was added to the column to cleave the 6xHis-SUMO tag. The cleaved protein was eluted and the fractions containing the TELSAM-MTase fusion protein were diluted to a final concentration of 50mM NaCl and loaded onto a 5ml HiTrap Q HP anion-exchange column (GE Healthcare). The protein eluted in the unbound fractions and was further purified by size exclusion chromatography on a HiLoad 16/600 Superdex 200 (GE Healthcare) column using 25mM Tris pH 8.3, 200mM KCl and 2mM TECP. All of the puri ed proteins were concentrated and stored in −80°C.

### Isothermal titration calorimetry (ITC).

The titrations were performed on a Microcal ITC_200_ instrument at 25°C with the standard 10μcals/s reference power and at 600rpm. The ligand SAM/SAH/SFG was loaded in the syringe (400μM) and titrated into 40μM of nsp14/nsp10 complex in the cell. Care was taken to ensure buffer match for the ligand and nsp14/nsp10 complex to eliminate heat from buffer mismatch. The titrations consisted of 15 injections of 2.5μl ligand solution at a rate of 0.5μl/s at 180s time intervals. An initial injection of 0.4μl was made and discarded during data analysis. The data were fit to a single binding site model using the Origin 7.0 software, supplied by MicroCal. All the experiments were repeated twice and average value reported.

### Crystallization of TELSAM-MTase protein with ligands.

Crystallization trials for all the constructs were carried out at 15mg/ml with 5-fold molar excess of the ligand (SAM, SAH and SFG). Initial screens were set up with Oryx Nano (Douglas Instruments) at 20°C using commercially available screens in a sitting drop format with 0.3μl of protein mixed with equal volume of reservoir solution. Among the three fusion constructs, only the fusion construct with a PAA linker produced initial hits. Initial crystals were observed in solutions containing 15% Reagent Alcohol, 0.2M Lithium Sulfate and 0.1M Sodium Citrate pH 5.5 in 2 days. The crystals were further optimized by varying both concentration of the Reagent Alcohol and also the pH of the buffer in hanging drop format using 1μl protein with 1μl reservoir. The crystals were cryoprotected in a step-wise manner with reservoir solutions containing 5–30% Glycerol and ash-cooled in liquid nitrogen. X-ray diffraction data were collected at the NSLS-II 17-ID-1 and 17-ID-2 beamlines at the Brookhaven National Laboratory (BNL) under cryogenic conditions.

The diffraction data were processed using DIALS and AIMLESS in the CCP4 suite^17,18 17,18^. The experimental data showed significant anisotropy and an anisotropic correction was performed using the STARANISO server (https://staraniso.globalphasing.org/cgi-bin/staraniso.cgi) with a surface threshold of I/σ(I) ≥ 1.2. The structure was solved by molecular replacement with Phaser-MR^19^ using the MTase domain of SARS-CoV (PDB-5C8T^2^) and TELSAM domain from PDB-7N1O^10^ as search models. Subsequent iterative manual building and refinement were performed with Coot and Phenix respectively^20,21^. Ligand restraint file for SFG was generated using eLBOW^22^ from the PHENIX suite. All molecular graphic figures were prepared by PyMOL (Schrödinger LLC).

### Accession Codes.

Atomic coordinates and structure factors for TELSAM-MTase-SAM, TELSAM-MTase-SAH and TELSAM-MTase-SFG have been deposited in the Protein Data Bank under the accession codes of 7TW7, 7TW8 and 7TW9, respectively.

## Supplementary Material

Supplement 1

## Figures and Tables

**Figure 1:**
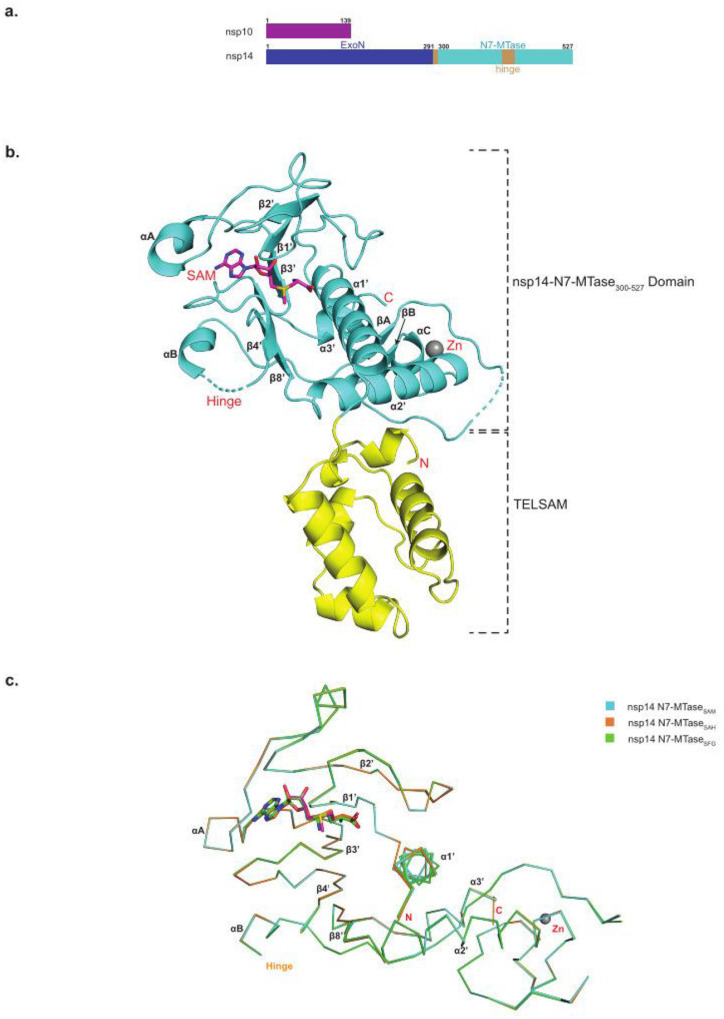
Overall structure. **a**. Domain organization of SARS-CoV-2 nsp14 and nsp10. **b**. The overall structure of TELSAM-MTase fusion in complex with SAM shown in a ribbon representation. The nsp14-N7-MTase domain and TELSAM are colored in cyan and yellow, respectively. The secondary structure elements for the N7-MTase domain are labeled. The residues not modeled in the structure are shown by dashed lines. A zinc ion (Zn) is shown as sphere and colored grey. **c**. C_α_ trace superposition of nsp14 N7-MTase_SAM_, nsp14 N7-MTase_SAH_ and nsp14 N7-MTase_SFG_.

**Figure 2:**
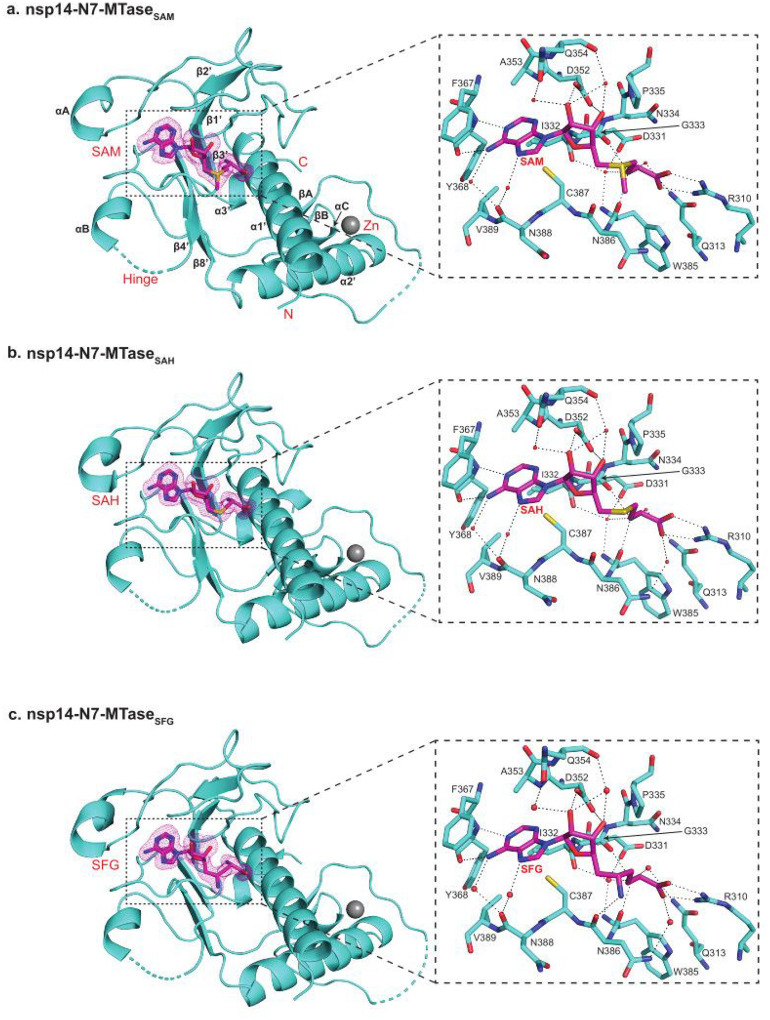
Details of SARS-CoV-2 nsp14-N7-MTase bound to ligands. **a**. Structure of nsp14 MTase domain bound to SAM (left), with a detailed view of the interactions between them (right). The F_o_-F_c_ difference electron density for SAM is shown in a pink mesh and contoured at 3σ. Hydrogen bonds between the MTase domain and SAM are depicted as dashed lines and the water molecules are shown as red sphere. **b**. Structure of nsp14 MTase bound to SAH (left), with a detailed view of the interactions between them (right). **c**. Structure of nsp14 MTase domain bound to SFG (left), with a detailed view of the interactions between them (right).

## References

[1] V’Kovski P., Kratzel A., Steiner S., Stalder H. & Thiel V. Nat Rev Microbiol 19, 155–170 (2021). 33116300 10.1038/s41579-020-00468-6 PMC7592455

[2] Ma Y. Proc Natl Acad Sci U S A 112, 9436–41 (2015). 26159422 10.1073/pnas.1508686112 PMC4522806

[3] Ogando N.S. Proc Natl Acad Sci U S A 118(2021).

[4] Lin S. Nucleic Acids Res 49, 5382–5392 (2021). 33956156 10.1093/nar/gkab320 PMC8136770

[5] Gorgulla C. ChemRxiv (2020).

[6] Devkota K. SLAS Discov 26, 1200–1211 (2021). 34192965 10.1177/24725552211026261 PMC8458670

[7] Liu C. Science 373, 1142–1146 (2021). 34315827 10.1126/science.abi9310 PMC9836006

[8] Yan L. Cell 184, 3474–3485 e11 (2021). 34143953 10.1016/j.cell.2021.05.033 PMC8142856

[9] Nauli S. Protein Sci 16, 2542–51 (2007). 17962407 10.1110/ps.073074207 PMC2211692

[10] Sarath Nawarathnage S.D. . bioRxiv (2021).

[11] Schubert H.L., Blumenthal R.M. & Cheng X. Trends Biochem Sci 28, 329–35 (2003). 12826405 10.1016/S0968-0004(03)00090-2 PMC2758044

[12] Ferron F. Proc Natl Acad Sci U S A 115, E162–E171 (2018). 29279395 10.1073/pnas.1718806115 PMC5777078

[13] Chen Y. J Virol 87, 6296–305 (2013). 23536667 10.1128/JVI.00061-13 PMC3648086

[14] Kaniskan H.U., Martini M.L. & Jin J. Chem Rev 118, 989–1068 (2018). 28338320 10.1021/acs.chemrev.6b00801 PMC5610952

[15] Ferreira de Freitas R., Ivanochko D. & Schapira M. Molecules 24(2019). 10.3390/molecules24244492 PMC6943651 31817960

[16] Lin H. ACS Med Chem Lett 10, 1033–1038 (2019). 31312404 10.1021/acsmedchemlett.9b00074 PMC6627734

[17] Evans P.R. & Murshudov G.N. Acta Crystallogr D Biol Crystallogr 69, 1204–14 (2013). 23793146 10.1107/S0907444913000061 PMC3689523

[18] Winter G. Acta Crystallogr D Struct Biol 74, 85–97 (2018). 29533234 10.1107/S2059798317017235 PMC5947772

[19] McCoy A.J. J Appl Crystallogr 40, 658–674 (2007). 19461840 10.1107/S0021889807021206 PMC2483472

[20] Emsley P. & Cowtan K. Acta Crystallogr D Biol Crystallogr 60, 2126–32 (2004). 15572765 10.1107/S0907444904019158

[21] Adams P.D. Acta Crystallogr D Biol Crystallogr 66, 213–21 (2010). 20124702 10.1107/S0907444909052925 PMC2815670

[22] Moriarty N.W., Grosse-Kunstleve R.W. & Adams P.D. Acta Crystallogr D Biol Crystallogr 65, 1074–80 (2009). 19770504 10.1107/S0907444909029436 PMC2748967

