# Supplementary material for: High resolution structures of the SARS-CoV-2 N7-methyltransferase inform therapeutic development
Source: Res Sq. 2022 Mar 8:rs.3.rs-1370473. Preprint. [Version 1] doi: 10.21203/rs.3.rs-1370473/v1 (PMC8923114; doi:10.21203/rs.3.rs-1370473/v1)
Supplement: Supplement 1 [file b8baa5cfebcb08945bebfd0c.pdf]

High resolution structures of the SARS-CoV-2 N7-methyltransferase inform therapeutic development

Jithesh Kottur and Aneel K. Aggarwal

Supplementary information

[1]Department of Pharmacological Sciences
Icahn School of Medicine at Mount Sinai
1425 Madison Avenue
New York, New York, USA

¶Correspondence: aneel.aggarwal@mssm.edu

Supplementary Table 1. Data collection and refinement statistics

	TELSAM-MTase-SAM	TELSAM-MTase-SAH	TELSAM-MTase-SFG
PDB ID	**7TW7**	**7TW8**	**7TW9**
Synchrotron Source	NSLS-II 17-ID-2	NSLS-II 17-ID-2	NSLS-II 17-ID-1
Detector	Dectris Eiger 16M	Dectris Eiger 16M	Eiger 9M pixel-array
Wavelength	0.97934	0.97934	0.92010
Space Group	P 6_5	P 6_5	P 6_5
Cell Dimensions			
a,b,c (Å)	108.8 108.8 48.5	109.2 109.2 48.7	109.1 109.1 48.6
α, β, γ (°)	90.00 90.00 120.00	90.00 90.00 120.00	90.00 90.00 120.00
Anisotropic truncation by STARANISO			
Diffraction limits from anisotropic analysis (Å)			
0.894 a* - 0.447 b*	1.99	1.76	1.83
b*	1.99	1.76	1.83
c*	1.52	1.50	1.38
Resolution range (Å)	94.20-1.62 (1.77-1.62)[a]	94.52-1.55 (1.68-1.55)[a]	54.52-1.41 (1.60-1.41)[a]
R_{merge} (%) (ellipsoidal)	7.2 (88.3)[a]	6.8 (87.1)[a]	10.8 (119.5)[a]
R_{pim} (%) (ellipsoidal)	3.9 (55.8)[a]	3.1 (53.4)[a]	4.9 (72.9)[a]
I/σ(I) (ellipsoidal)	12.4 (2.0)[a]	13.4 (1.7)[a]	9.2 (2.2)[a]
$CC_{1/2}$ (ellipsoidal)	99.9 (58.1)[a]	99.9 (76.2)[a]	99.6 (49.3)[a]
Completeness (Spherical) (%)	66.2 (14.8)[a]	75.3 (17.0)[a]	56.1 (8.3)[a]
Completeness (ellipsoidal) (%)	94.3 (76.0)[a]	95.4 (71.6)[a]	94.6 (71.3)[a]
Redundancy	8.1 (5.8)[a]	10.6 (5.9)[a]	11.1 (7.1)[a]
Refinement			
Resolution (Å)	36.2-1.62	36.2-1.55	36.3-1.41
R_{work} (%)/R_{free} (%)	17.8/21.6	17.7/20.6	19.2/23.2
No. of Reflections all/ R-free (%)	27531/1352 (4.91%)	36499/1737 (4.76%)	36850/1833 (4.97%)
No. of non-hydrogen atoms			
Protein	2141	2135	2145
Ligand (SAM/others)	27/-	26/-	27/20
Water	342	363	377
Protein residues	262	265	261
Average B-factors [$Å^2$]			
Protein	23.89	25.06	18.74
Ligand (SAM/others)	18.82/-	18.46/-	12.58/38.57
Water	32.42	38.31	32.86
Ramachandran Plot			
Ramachandran favored (%)	97.27	97.29	98.04
Ramachandran allowed (%)	2.73	2.71	1.96
Ramachandran outliers (%)	0.00	0.00	0.00
R.M.S. deviations			
Bond lengths (Å)	0.015	0.004	0.008
Bond angles (°)	1.21	0.83	0.73

[a]Highest resolution shell is shown in parentheses.

Supplementary Table 2. Thermodynamic binding parameters of SAM, SAH, and SFG to nsp14/nsp10 complex

Ligand	*n* Stoichiometry	K_D (μM)	ΔG (Kcal mol^{-1})	ΔH (Kcal mol^{-1})	−TΔS (Kcal mol^{-1})
SAM	0.94±0.02	5.70 ± 0.6	-7.22 ± 0.3	-8.2 ± 0.3	0.98
SAH	0.92±0.01	0.30 ± 0.02	-8.86 ± 0.2	-17 ± 0.2	8.14
SFG	0.97±0.02	4.40 ± 0.59	-7.21 ± 0.3	-8.4 ± 0.3	1.19

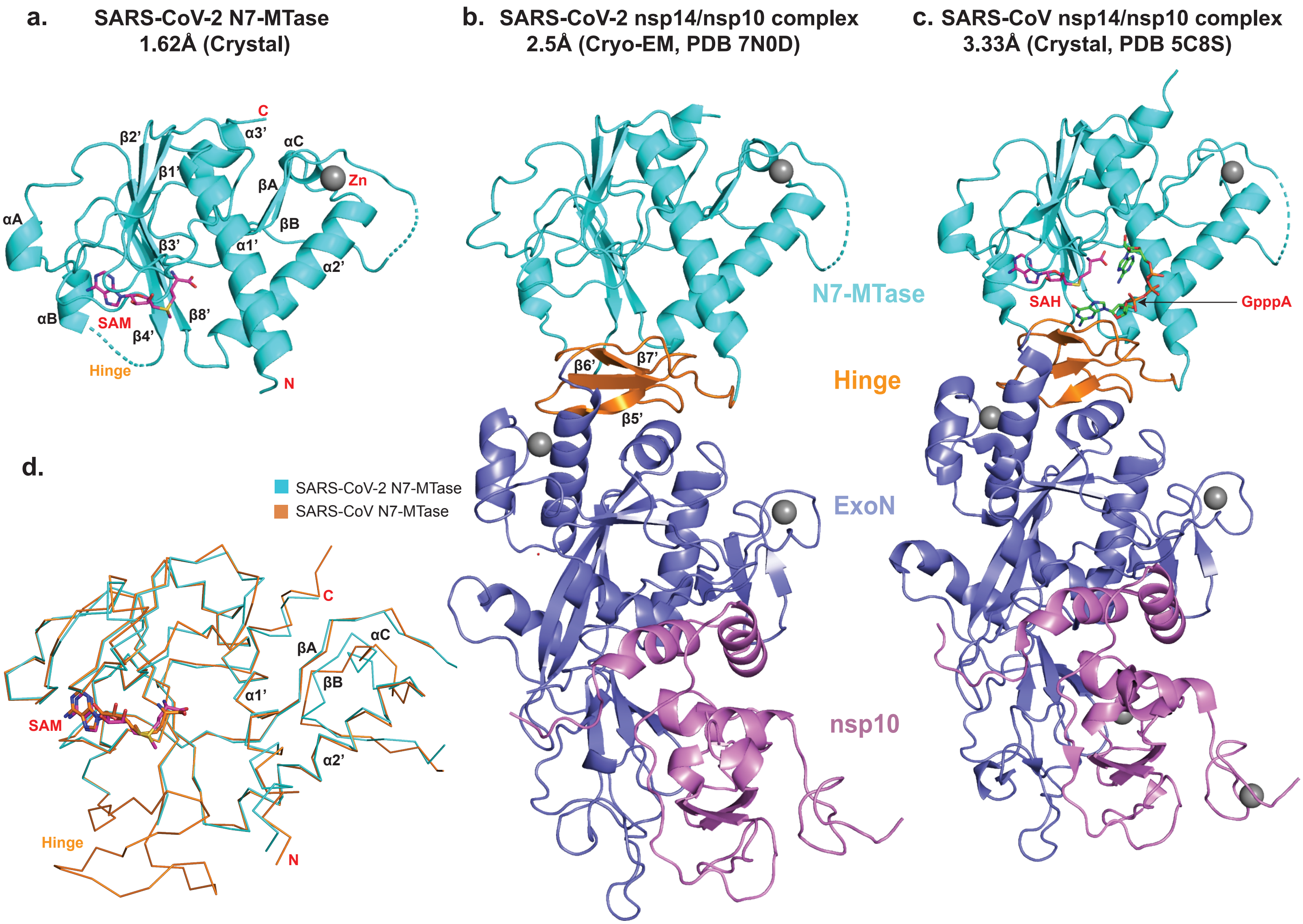

Supplementary Figure 1. Structural comparison of nsp14-N7-MTases. a. Structure of SARS-CoV-2 nsp14-N7-MTaseSAM complex. **b**. Cryo-EM structure of SARS-CoV-2 nsp14/nsp10 heterodimer (PDB:7N0D, chain H). **c**. Crystal structure of SARS-CoV nsp14/nsp10GpppA:SAH complex (PDB:5C8S, chain B). The nsp14 MTase core, ExoN domain, and the hinge region are colored cyan, slate blue and orange, respectively. The nsp10 subunit is colored purple. **d**. Cα trace superposition of the MTase domains of SARS-CoV-2 (cyan) and SARS-CoV (PDB: 5C8T, chain B, orange). The bound SAM is also shown.

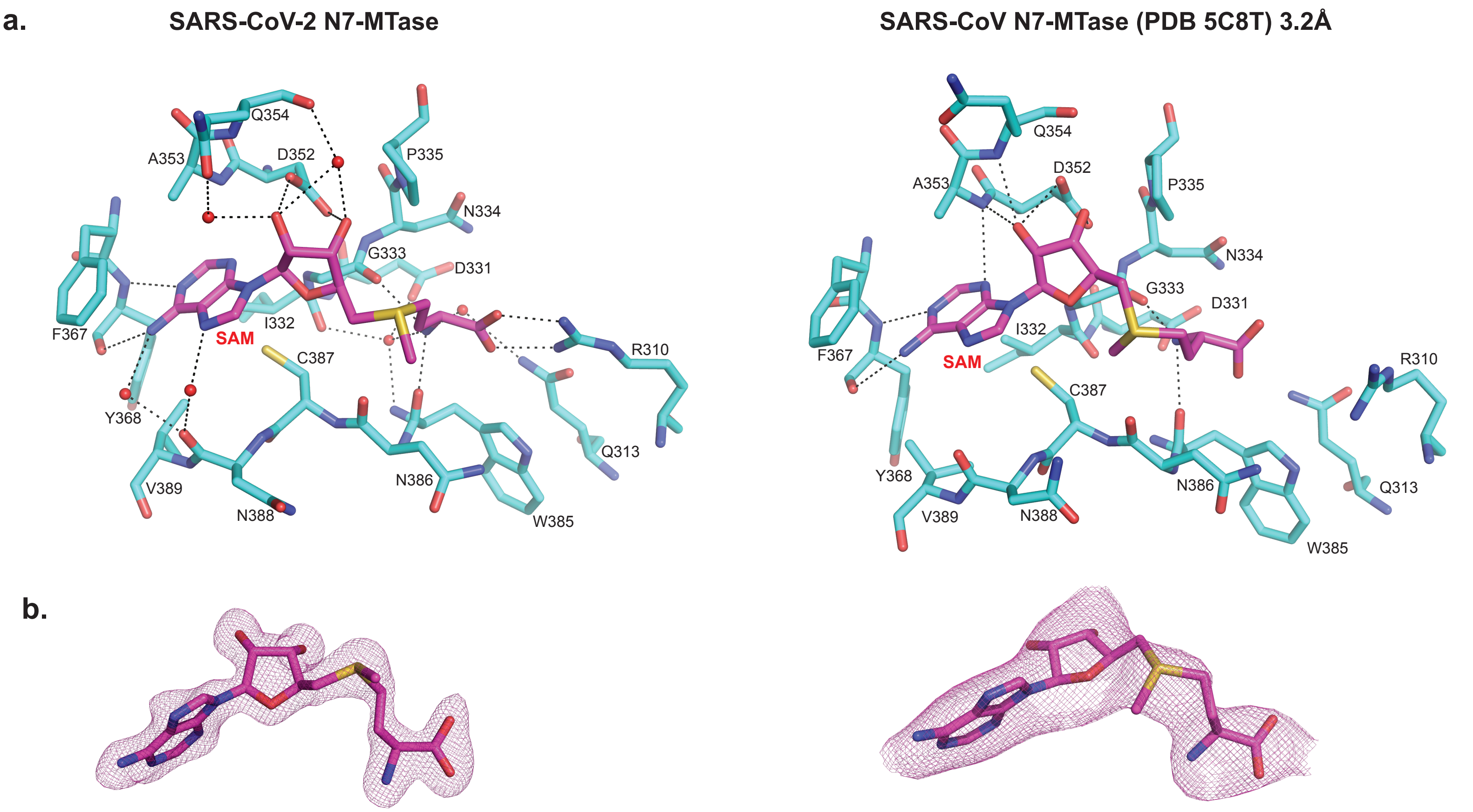

Supplementary Figure 2. Comparison of atomic binding details of nsp14-N7-MTase. a. Atomic binding details of SARS-CoV-2 nsp14-N7-MTase (left) and previously reported SARS-CoV nsp14/nsp10 (PDB: 5C8T, chain B, right) with bound SAM. Hydrogen bonds are depicted as dashed lines and water molecules are shown as red spheres. **b**. Fo-Fc difference density map for SAM in the SARS-Cov-2 N7-MTaseSAM (left) and SARS-CoV nsp14/10SAM structures (right) contoured at 3σ.

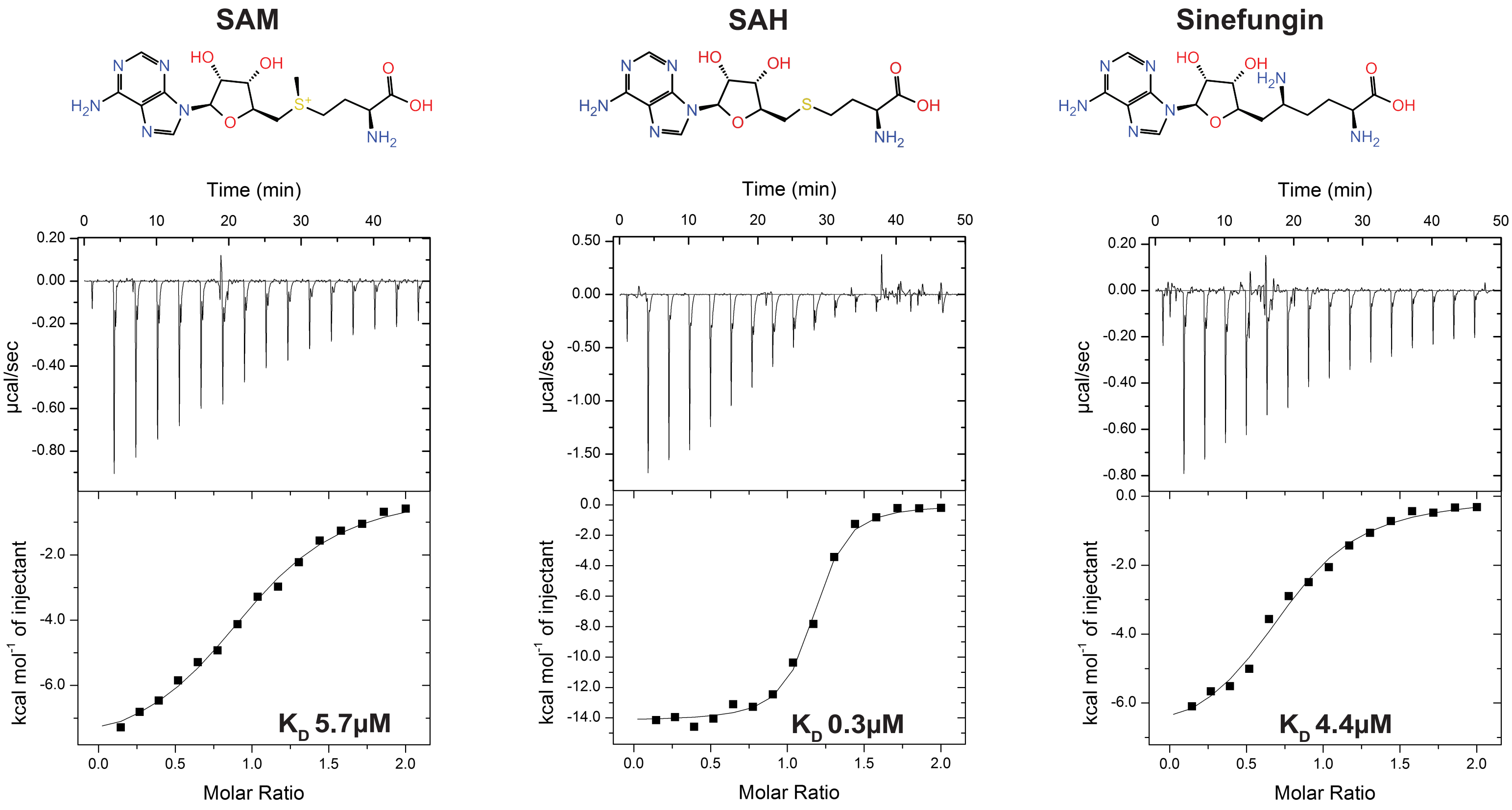

Supplementary Figure 3. ITC analysis of nsp14/10 complex with SAM, SAH and SFG. ITC titration data for SAM (left), SAH (middle) and SFG (right) into nsp14/nsp10 complex are shown. The equilibrium dissociation constants (KD) were derived from the resulting binding isotherms. The chemical structures of each ligand are also shown.

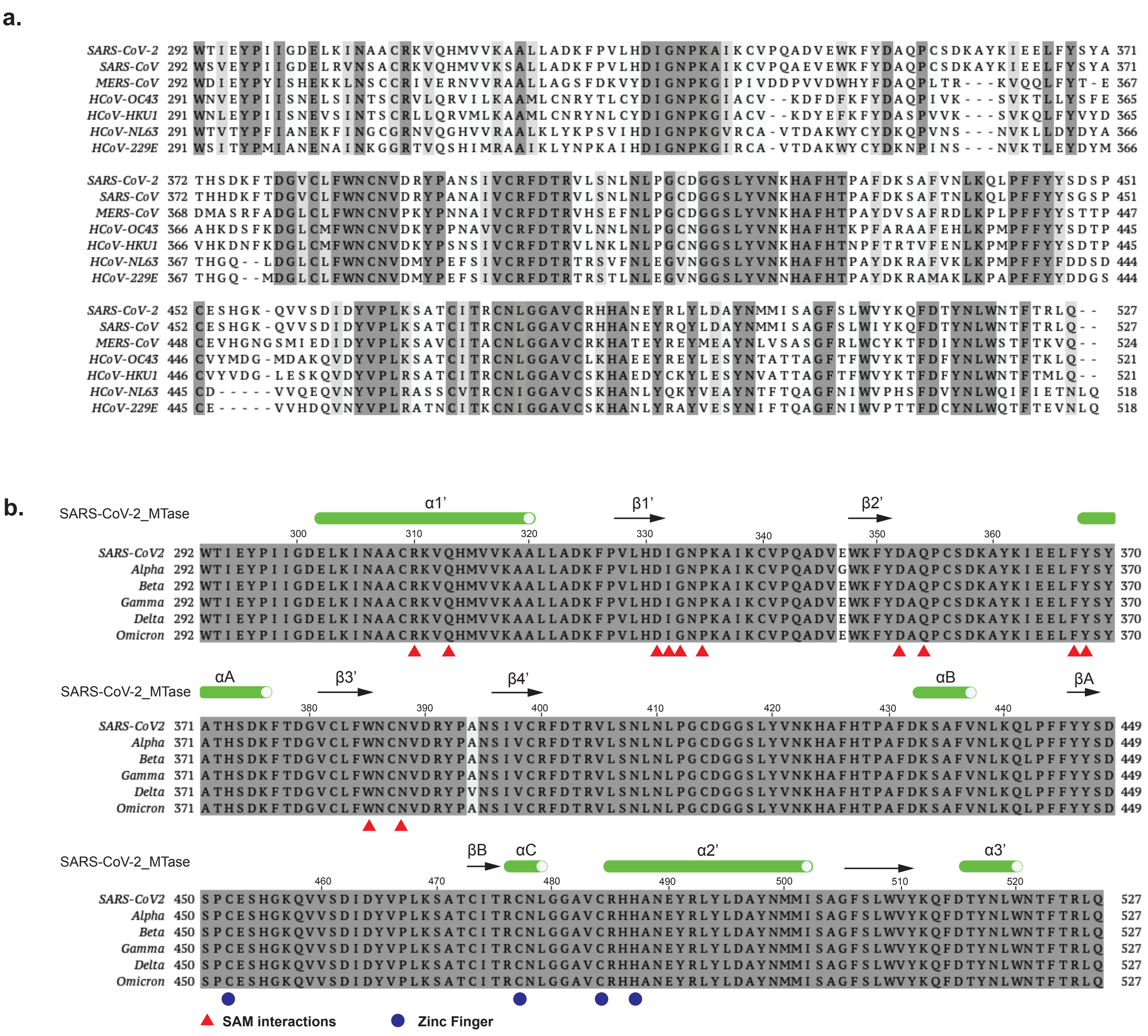

Supplementary Figure 4. Sequence comparison. a. Sequence alignment of the nsp14 N7-MTase domain from 7 pathogenic coronaviruses. The identical and similar residues are highlighted in dark grey and light grey, respectively. **b**. Sequence alignment of the nsp14 N7-MTase domain of SARS-CoV-2 and its variants. The secondary structure elements are shown above the sequence alignment. Red triangles highlight residues that interact with SAM. Blue circles highlight residues that coordinate the zinc ion.
